# *NF1*-Dependent Transcriptome Regulation in the Melanocyte Lineage and in Melanoma

**DOI:** 10.3390/jcm10153350

**Published:** 2021-07-29

**Authors:** Lionel Larribère, Jochen Utikal

**Affiliations:** 1 Skin Cancer Unit, German Cancer Research Center (DKFZ), 69120 Heidelberg, Germany; j.utikal@dkfz.de; 2 Department of Dermatology, Venereology and Allergology, University Medical Center Mannheim, Ruprecht-Karl University of Heidelberg, 68167 Mannheim, Germany

**Keywords:** melanoblast, melanoma, NF1, transcriptome, RNAseq

## Abstract

The precise role played by the tumor suppressor gene *NF1* in melanocyte biology and during the transformation into melanoma is not completely understood. In particular, understanding the interaction during melanocyte development between *NF1* and key signaling pathways, which are known to be reactivated in advanced melanoma, is still under investigation. Here, we used RNAseq datasets from either situation to better understand the transcriptomic regulation mediated by an *NF1* partial loss of function. We found that *NF1* mutations had a differential impact on pluripotency and on melanoblast differentiation. In addition, major signaling pathways such as VEGF, senescence/secretome, endothelin, and cAMP/PKA are likely to be upregulated upon *NF1* loss of function in both melanoblasts and metastatic melanoma. In sum, these data bring new light on the transcriptome regulation of the *NF1*-mutated melanoma subgroup and will help improve the possibilities for specific treatment.

## 1. Introduction

The implication of the *NF1*-encoded protein neurofibromin (NF1) in melanocyte biology has been investigated for more than 20 years. Despite its ubiquitous expression, NF1 has been described as regulating lineage-specific mechanisms by interacting with melanocyte-associated genes such as MITF or cKIT [1]. Indeed, *NF1* germline mutations induce neurofibromatosis type 1 syndrome, manifestations of which include defects in neural crest-derived cells such as melanocytes [2]. The embryological origin of these defects was previously described in *Nf1* mouse models. In fact, *Nf1*^+/−^ or *Nf1*^−/−^ mice in which mutations were specifically induced in the melanocyte lineage showed hyperpigmentation [3,4]. Moreover, a partial rescue of the belly spot phenotype in *Mitf^Mi-wh^*^/+^ or *cKit^W^*^/+^ mice was observed when the latter were crossed with *Nf1*^+/−^ mice [5,6]. These “café-au-lait” macules, which are benign melanocytic lesions, can be observed in almost all patients [7,8], and the cells located in the macules usually carry a second somatic mutation on the wildtype *NF1* allele suggesting a selective advantage to the cell subpopulation [9].

The main described function of NF1 is a GTPase activity which inactivates RAS [10], and RAS deregulation in the melanocyte lineage is known to lead to pigmentation defects [11]. This phenotype can be explained at least in part by the fact that pigmentation’s main regulator, MITF, can be phosphorylated by an active RAS-ERK pathway [12]. NF1 can also be phosphorylated by the protein kinase A (PKA), whose pathway is activated by cAMP and plays an important role during melanogenesis [13]. Another NF1 function is to interact with the cytoskeleton structures in order to control lamellopodia formation and actin polymerization. These processes are mainly regulated by RAC1 signaling, and it was recently observed that variable *NF1* expression levels changed the cell migration rate of melanoblasts in an RAC1-dependent manner [14]. Moreover, microtubule network fluctuation as well as FAK activity may also be influenced by NF1 [15,16,17]. Furthermore, NF1’s interaction with cKIT signaling leads to a dysregulation of the melanoblast migration [6]. 

Interestingly, the migration and invasive phenotype of melanoblasts during development closely resemble the aggressive behavior of melanoma metastases [18,19,20]. The cell plasticity of lineage-committed melanoblasts is also observed in certain clones of melanoma tumors which have undergone dedifferentiation and which are usually refractory to treatments [21,22]. Recently, miRNAs identified in a human model of melanoblasts were described as potential drivers of melanoma development [23]. Malignant melanoma has been classified in four subgroups according to its main genetic drivers by The Cancer Genome Atlas: mutant *BRAF*, mutant *NRAS*, mutant *NF1*, or triple wild-type tumors [24]. In general, mutant *NF1* melanoma contain WT *BRAF* and WT *NRAS*, and rather carry comutations in the so-called RASopathy genes (*PTPN11* or *RASA2*). These tumors typically appear on chronically sun-exposed skin or in older individuals [25,26]. However, *NF1* mutations can sometime appear concurrently with *BRAF* or *NRAS* mutations. Indeed, a classification in three subgroups has also been suggested: mutant *BRAF*, mutant *NRAS*, non-*BRAF*mut/non-*NRAS*mut [27]. These co-occurring mutations lead to a higher RAS GTPase activity and resistance to BRAF inhibitors [28]. For example, the treatment of melanoma cells with the combination of BRAF inhibitor and MEK inhibitor showed either a loss of *NF1* expression or an acquired mutation [29]. Mutations cooperation between *NF1* and *BRAF* was also described in melanocytic nevi to explain the bypass of oncogene-induced senescence (OIS) and the progression to transformed melanoma [30]. Nevertheless, although 15% of sporadic melanoma bear *NF1* somatic mutations, no targeted treatment has been developed for this patient’s subgroup, mainly due to the complexity of the protein.

In this report we intended to analyze the NF1-controlled transcriptome and identify regulated signaling pathways in two independent models: *NF1*^+/−^ induced pluripotent stem cell-derived melanoblasts and their late stage transformed counterpart, *NF1*^+/−^ metastatic melanoma.

## 2. Materials and Methods

### 2.1. Cell Lines

Three human induced pluripotent stem cell (hiPSC) lines were generated from fibroblasts obtained from skin biopsies of two patients with Neurofibromatosis Type 1 carrying *NF1* mutations (University of Ulm, Ulm, Germany) (one clone from the first patient and two clones from the second patient), and three hiPSC lines were generated from three healthy donors (University Medical Center Mannheim, Mannheim, Germany) (one clone from each patient) according to the ethical regulation. Patients with *NF1* mutations did not present melanoma tumors. The reprogramming protocol was already described by Larribere et al. [31]. Melanoblasts were derived from either *NF1*-mutated or *NF1*-wildtype hiPSC lines following a previously published protocol [1].

### 2.2. RNA Sequencing and Analysis

Briefly, total RNA was isolated with an RNeasy kit (Qiagen, Hilden, Germany), and DNase I digestion was performed to remove genomic DNA. Illumina sequencing libraries were prepared using the TruSeq Stranded mRNA Library Prep Kit (Illumina, Eindhoven, Netherland) according to the manufacturer’s protocol. Briefly, poly(A)+ RNA was purified from 500 ng of total RNA using oligo(dT) beads, fragmented to a median insert length of 155 bp, and converted to cDNA. The ds cDNA fragments were then end-repaired, adenylated on the 3′ end, adapter-ligated, and amplified with 15 cycles of PCR. The libraries were quantified using a Qubit ds DNA HS Assay kit (Life Technologies-Invitrogen, Darmstadt, Germany) and validated on an Agilent 4200 TapeStation System (Agilent technologies, Waldbronn, Germany). Based on the Qubit quantification and sizing analysis, multiplexed sequencing libraries were normalized, pooled (4plex), and sequenced on HiSeq 4000 single-read 50 bp with a final concentration of 250 pM (spiked with 1% PhiX control). The tool bcl2fastq (version 2.20.0.422, Illumina, Eindhoven, Netherland) was used for generating the fastq files and demultiplexing. 

Gene set enrichment analyses were performed with Ingenuity Pathway Analysis (IPA) (Ingenuity^®^ Systems, Redwood City, CA, USA, www.ingenuity.com) using a Log2-threshold = 2, *p*-value < 0.05.

### 2.3. Data from cBioportal Database

The melanoma sample’s genomic dataset originated from the Skin Cutaneous Melanoma study (TCGA, Firehose Legacy) in the cBioportal database (Memorial Sloan Kettering Cancer Center, New York, NY, USA). Selected metastatic melanoma samples included patients with distant metastases or metastases in regional lymph nodes, as established by the American Joint Committee on Cancer (AJCC). These samples were divided in two groups according to a high (*NF1*-high, *n* = 25) or low (*NF1*-low, *n* = 26) expression level of *NF1* with a z-score threshold of 1. Accordingly, these filtered samples bore either loss-of-function SNPs or heterozygous deletions (*NF1*-low) or no *NF1* mutations (*NF1*-high).

The genomic dataset from the Cancer Cell Line Encyclopedia (Broad, 2019) contained a large range of tumor entities including melanoma cell lines (about 6%). As before, these samples were divided in two groups according to a high (*NF1*-high, *n* = 64) or low (*NF1*-low, *n* = 61) *NF1* mRNA expression (z-score relative to diploid samples, threshold = 1). 

## 3. Results and Discussion

### 3.1. NF1 Mutations Have Differential Impact on Pluripotency and on Differentiation

**Figure 1:**
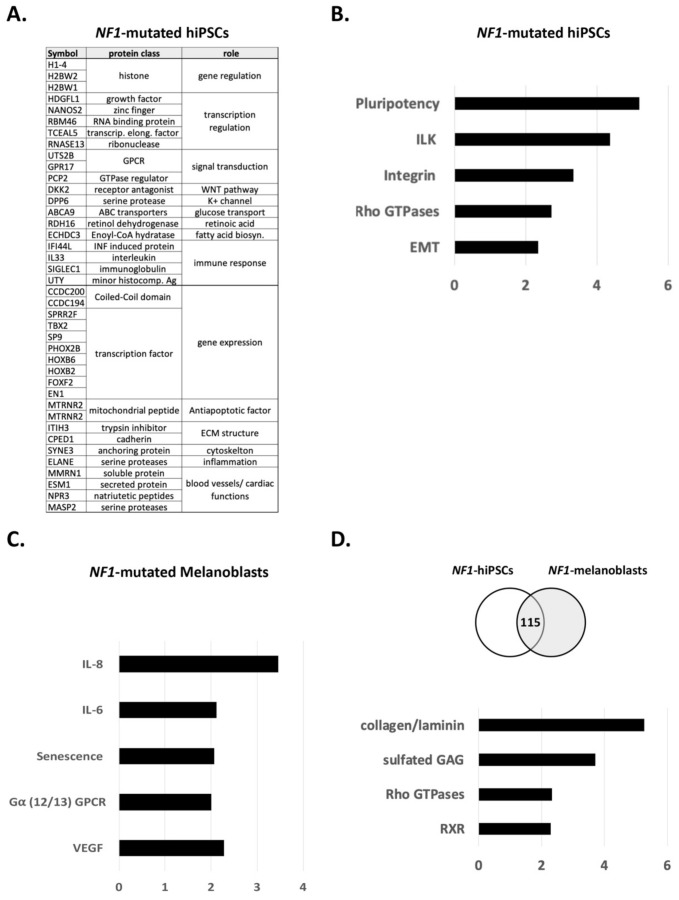
*NF1* mutations have a differential impact on pluripotency and on differentiation. (**A**) Top 20 regulated genes in *NF1*-mutated hiPSCs compared to *NF1*-WT hiPSCs. (**B**) Main regulated signaling pathways in *NF1*-mutated hiPSCs compared to *NF1*-WT hiPSCs. (**C**) Main regulated signaling pathways in *NF1*-mutated melanoblasts compared to *NF1*-WT melanoblasts. (**D**) Common regulated signaling pathways in *NF1*-mutated hiPSCs and in *NF1*-mutated melanoblasts.

The transcriptome of NF1-mutated human induced pluripotent stem cells (hiPSCs) was analyzed by RNA sequencing and compared to wildtype control hiPSCs, in order to specifically study the impact of NF1 mutations during reprogramming. The top 20 regulated genes are represented in Figure 1A. Among these genes, one can find regulators of DNA replication (histones), transcription and gene regulation (transcription factors), genes involved in cellular (cytoskeleton) and extracellular matrix structures (cadherin). Genes were also involved in intracellular signal transduction (G protein-coupled receptor (GPCR) and GTPases) or in ion or glucose transport. Genes involved in the WNT, Retinoic Acid, or Fatty Acid Biosynthesis pathways were also reported. We observed genes involved in biological functions such as apoptosis, inflammation, or immune response. Finally, many genes were involved in blood vessels or cardiac function, which is in line with the observed main defects leading to the short survival of Nf1^−/−^ mice [32].

A pathway analysis of the most regulated genes with the IPA software resulted in the following signalings (Figure 1B). Genes involved in pluripotency such as SMAD6, TGFB, WNT2B, WNT3, WNT5A were retrieved, although no phenotypic differences have been observed between NF1-mutated hiPSCs and NF1-WT hiPSCs [31]. These data may argue for a role of WNT and SMAD pathways during NF1-mutated stem cell reprogramming that is less important than other pathways such as FGFR2. Indeed, FGFR signaling controls cell survival, proliferation, embryonic development, and organogenesis by activating MAPK, PI3K/AKT, PLCγ, and STAT pathways [33]. These pathways are also described as interacting with WNT and TGFβ and could in this situation play redundant functions. ILK and more generally the integrin pathway promote several cellular functions such as adhesion, growth, and migration. The involvement of NF1 in migration has for example been established [14,17]. Rho GTPases belong to a subfamily of the Ras superfamily and are therefore expected to interact with NF1 [34]. This study from Upadhyaya et al., already discussed the role of the Rho GTPase pathway in NF1 tumorigenesis. Epithelial-to-mesenchymal transition (EMT) is a biological process that occurs during embryogenesis but also in cancer metastases. During reprogramming, cells will also undergo sequential EMT and mesenchymal-to-epithelial transition (MET), the latter being required for pluripotency acquisition [35]. Pathways activated by RAS (and likely by NF1 loss) have been described as playing a crucial role during EMT and MET. For instance, Arima et al., described the loss of NF1-activated EMT-related signalings and, additionally, that an excessive mesenchymal phenotype may play a role in the development of NF1-associated neurofibromas [36]. 

Next, we analyzed the transcriptome of melanoblasts differentiated from NF1-mutated hiPSCs and compared it to that of wildtype control melanoblasts [1]. A similar IPA pathway analysis presented the following results (Figure 1C). In contrast to NF1-mutated hiPSCs, regulated genes in NF1-mutated melanoblasts were mainly involved in the senescence, IL-6/8, VEGF, and GPCR pathways. Interestingly, oncogene-induced senescence (OIS) generated by the loss of NF1 expression was described in melanoma cells before [30] and was also observed specifically in melanocytes located in the NF1-associated “café-au-lait” macules [31]. OIS leads to the secretion of a panel of cytokines in the extracellular environment, which includes IL-6/8 acting as a paracrine activation loop, as previously described [37,38]. These data bring additional confirmation of NF1′s role in the senescence of melanocytes precursors and in the regulation of the secretome. The involvement of NF1 during melanocyte differentiation was reproduced in a study using NF1^+/−^ embryonic pluripotent stem cells [39]. This NF1 model allowed one to link NF1 mutations to the hyperpigmentation of melanocytes and to replicate the loss of heterozygosity specifically in CALMs.

Although the VEGF pathway does not play a major role in melanoblast differentiation, an essential role of NF1 in controlling endothelial cell proliferation was described [40]. Indeed, the authors of this study showed a reduction of NF1 expression upon VEGF stimulation and the consequent activation of MAPK and PI3K-AKT pathways. Finally, the activation of the GPCR superfamily was observed. In particular, the coupled G_α12/13_ protein subunit regulates cellular proliferation, movement, and morphology and is also involved in cancer [41]. For example, after the binding of endothelin to its receptor EDNRA, coupled G_αq_/G_α11_ and G_α12_/G_α13_ proteins are activated [42]. However, the function of neural crest cells and their derived melanoblasts is dependent on EDNRB activation and on the involvement of coupled G_αi_/G_α0_, G_αq_/G_α11_, or G_α13_ subunits. The endothelin signaling leads to the activation of phospholipase Cβ, inhibition of adenyl cyclase, activation of plasma membrane Ca^2+^ channels, and activation of nonreceptor tyrosine kinases [43,44]. Of note, genes encoding for some members of associated G proteins (GNAS, GNAQ, and GNA11) have been described as being mutated in tumors and as being oncogenic drivers of uveal melanoma [45]. It is interesting to retrieve the endothelin receptor coupled G protein since the complex relationship between endothelin signaling and NF1 has already been discussed based on pigmentation transgenic mice models [4]. Indeed, NF1 is likely to play different roles in skin and fur pigmentation [46].

We also analyzed common NF1-induced pathway regulation in hiPSCs and in melanoblasts (Figure 1D). Overlapping regulated genes belong to signalings involved in general cellular functions such as cell differentiation, migration, and adhesion, confirming the pleiotropic role of NF1. For example, collagen, laminin, or sulfated glycosaminoglycans families, which are expressed at the cell membrane and in the extracellular matrix, or the actin network were commonly regulated. The Rho GTPase family, which was described above as being potentially associated to pluripotency function, is also likely to be involved in more general cellular functions concerning Nf1-mutated melanoblasts [34]. Last, the RXR family of nuclear receptors could be regulated by NF1 signaling. For instance, similar ventricular myocyte defects were observed in *Rxrα*^−/−^ mouse embryos and in embryos with homozygous mutations on *Nf1* [47].

### 3.2. Role of NF1 in the Gene’s Regulation of Melanoblasts and Melanoma

**Figure 2:**
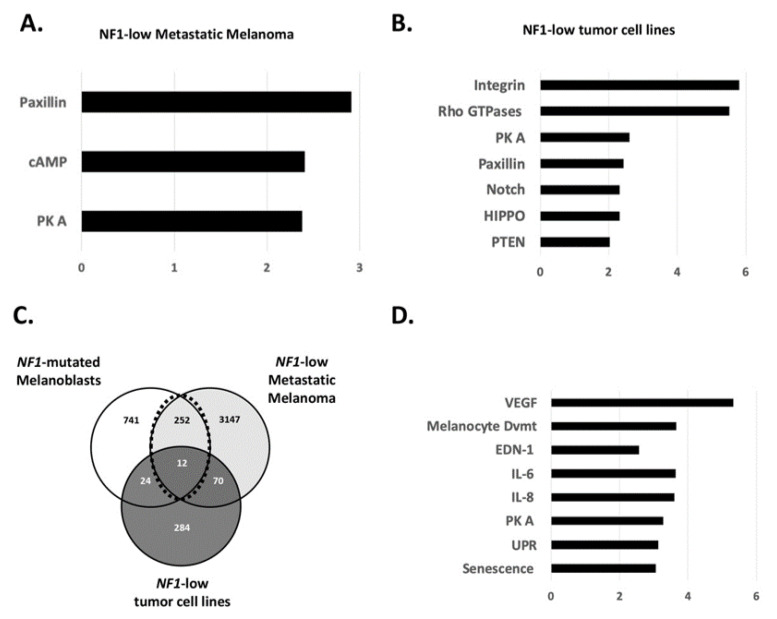
Role of NF1 in the gene’s regulation of melanoblasts and melanoma. (**A**) Main regulated signaling pathways in *NF1*-low expressing metastatic melanoma. (**B**) Main regulated signaling pathways in *NF1*-low expressing tumor cell lines. (**C**) Venn diagram including *NF1*-low expressing metastatic melanoma, *NF1*-mutated melanoblasts, and *NF1*-low expressing tumor cell lines. (**D**) Common regulated signaling pathways in *NF1*-mutated melanoblasts and *NF1*-low expressing metastatic melanoma.

Next, we used the RNAseq dataset of the TCGA Firehose melanoma cohort from the publicly available cBioportal database [48,49]. 161 metastatic melanoma samples were divided in two groups according to a high or low expression level of NF1 mRNA (z-score threshold of 1). Surprisingly, the number of regulated genes between the two groups was not very high (*n* = 229, log2-FC threshold: 2). Consequently, a discrete number of pathways appeared to be overrepresented in NF1-low expressing metastatic melanoma samples: Paxillin, cAMP, and PKA (Figure 2A). The influence of the cAMP/PKA pathway in melanoma development has been described. The MC1R/cAMP pathway controls UV-mediated melanin synthesis, and certain mutations in the MC1R gene lead to pigment defects in people with red hair and fair skin, therefore increasing the risk of melanoma [50]. More recently, structural biology studies on the MC1R/cAMP signaling pathway have focused on its mechanisms for enhancing genomic stability, suggesting pharmacologic opportunities to reduce melanoma risk [51]. The role of the cAMP pathway in melanoma treatment resistance has also been described before. Systematic gain-of-function resistance studies have revealed a cAMP-dependent melanocytic signaling network associated with drug resistance, including GPCR, adenyl cyclase, PKA, and cAMP response element binding protein (CREB) [52]. More recently, an alternative activation of the cAMP pathway in melanocytes has been shown downstream of the G protein-coupled estrogen receptor. This activation led to melanoma cell vulnerability to immunotherapy [53]. However, the specific role of NF1 in melanoma treatment resistance is still unclear. Earlier sensitivity assays of MEK inhibitors in BRAF-WT, NRAS-WT, and NF1-mutated melanoma did not show a high correlation [25,54]. More experimental studies focusing on the exact role of NF1 and cAMP in the context of treatment resistance are needed. Paxillin is involved in cellular adhesion to the extracellular matrix (via binding to focal adhesion kinases, FAK) and therefore contributes to migration. A defect in the paxillin expression can provoke cancer progression [55,56]. Along this line, NF1 is described as interacting with FAK, and its deregulation influences cellular migration [17]. Recently, the loss of NF1 in melanoma cells led to increased migration and metastases formation in vivo [14]. 

Similarly, we addressed the same question in the panel of tumor cell lines from the Cancer Cell Line Encyclopedia (CCLE). We filtered NF1-low and -high expressing cell lines in cBioportal and compared the two groups for gene regulation. The PKA and Paxillin pathways were redundant with the metastatic melanoma samples (Figure 2B). Integrins and Rho GTPases were also regulated, as we observed in hiPSCs samples, arguing for a more general role of NF1 in proliferating cells. In addition, three other pathways known to play crucial roles in transformation and tumor progression were found: Notch, Hippo, and PTEN. Evidence for an NF1 role in the transforming activity of the Notch pathway has been gathered in the context of Schwann cells transformation [57]. The modulation of the Hippo pathway has been reported to be mediated by NF1 loss in neurofibroma [58]. Despite being major tumor suppressors, PTEN and NF1 may not play redundant functions. Indeed, the concurrent loss of PTEN and NF1 in different tumor settings has been reported [59,60].

Last, we wanted to focus on the 263 genes which were regulated in both NF1-mutated melanoblasts and in NF1-low expressing metastatic melanoma (Figure 2C) because genes involved in melanocyte development are likely to be reactivated during melanoma progression [23,61]. The genes likely regulated by NF1 were involved in common signalings. Again, we found IL-6/8 and senescence/UPR signalings pathways (Figure 2D). Indeed, the Unfolded Protein Response (UPR) pathway plays a role in the cellular equilibrium between senescence and apoptosis [62]. Although not deeply investigated, NF1 could play a role in this pathway. In an Nf1-mutated mouse model of malignant peripheral nerve sheath tumors (MPNSTs), increased levels of UPR-associated genes (p-eIF2α, XPB1s, and GRP78) have been shown [63]. VEGF signaling, which we found regulated in NF1-mutated melanoblasts, is also associated with melanoma progression [64]. The endothelin pathway seems to play a role in melanoma. Recently, it was described how endothelin signaling promoted melanoma tumorigenesis driven by constitutively active GNAQ [65]. Additionally, the inhibition of EDNRB receptor signaling synergizes with MAPK pathway inhibitors in BRAF-mutated melanoma [66]. 

Finally, we mentioned above the redundant role of cAMP/PKA signaling during melanogenesis [13]. The implication of NF1 in this pathway has also been studied. Loss of NF1 leads to the activation of the cAMP/PKA pathway, which consequently phosphorylates the melanocyte-associated transcription factor MITF [46]. Such post-translational modifications of MITF leading to varying expression levels have been described in tumor cell subpopulations which respond differently to melanoma treatments [67,68]. 

## 4. Conclusions

In conclusion, we here describe transcriptomic signatures found not only in metastatic melanoma tumor cells but also in melanocyte progenitors, melanoblasts, in the case of an *NF1* partial loss of function. These signatures included the activation of VEGF, senescence/secretome, endothelin, and cAMP/PKA signaling pathways. More clinical research focusing on *NF1*-mutated melanoma, one of the four molecular subgroups classified by The Cancer Genome Atlas, is expected since no efficient targeted therapy has so far been available, unlike for the *BRAF*-mutated subgroup (BRAF/MEK inhibitors) or *NRAS*-mutated subgroup (MEK inhibitors clinical trials).

## Data Availability

RNAseq data are available upon request.
